# A highly sensitive amplicon sequencing workflow for genomic surveillance of Usutu virus

**DOI:** 10.1186/s12985-026-03251-w

**Published:** 2026-07-10

**Authors:** Gábor Endre Tóth, Anna Nagy, Jaime A. Costales, M. Alejandra Camacho, Santiago F. Burneo, Marike Petersen, Alexandra Bialonski, Heike Baum, Balázs Horváth, Anna Heitmann, Renke Lühken, Michael Schmidt, Jonas Schmidt-Chanasit, Zsófia Tauber, Dániel Cadar

**Affiliations:** 1 Virus Metagenomics and Evolution Group, Bernhard Nocht Institute for Tropical Medicine, Hamburg, Germany; 2 Department of Arbovirology and Entomology, Bernhard Nocht Institute for Tropical Medicine, Hamburg, Germany; 3 National Reference Laboratory for Viral Zoonoses, National Center for Public Health and Pharmacy, Budapest, Hungary; 4 Centro de Investigación para la Salud en América Latina, Pontificia Universidad Católica del Ecuador, Quito, Ecuador; 5 Museo de Zoología, Pontificia Universidad Católica del Ecuador, Quito, Ecuador; 6 Vector Control Group, Bernhard Nocht Institute for Tropical Medicine, Hamburg, Germany; 7 Institute of Transfusion Medicine and Immunohematology, German Red Cross Blood Transfusion Service Baden-Württemberg-Hessen, Frankfurt am Main, Germany; 8 Faculty of Mathematics, Informatics and Natural Sciences, University of Hamburg, Hamburg, Germany; 9 School of Biomedical Sciences, University of Plymouth, Plymouth, UK

**Keywords:** Usutu virus, Blood donors, Amplicon sequencing, Genomic surveillance, Transfusion safety, Next-generation sequencing, Metagenomics

## Abstract

Genomic surveillance of Usutu virus (USUV) in blood donors is hampered by extremely low viral loads, which usually prevent reliable genome sequencing. We developed and validated a tiled amplicon-based sequencing protocol optimized for low-titer samples. Serial dilutions of four phylogenetically distinct USUV lineages showed ≥ 95% genome recovery above 100 RNA copies/µL and 65–98% recovery between 3 and 100 copies/µL. We applied the method to 27 USUV-positive blood donors from Germany (median 1.70 copies/µL), achieving lineage assignment in 74% and ≥ 70% genome coverage in 63% of samples. This approach enables routine genomic surveillance of USUV in blood donors.

**Supplementary Information:**

The online version contains supplementary material available at 10.1186/s12985-026-03251-w.

## Background

Usutu virus (USUV, *Orthoflavivirus usutuense*) is a mosquito-borne orthoflavivirus that has been circulating in Europe since its first detection in Austria in 2001 and retrospectively identified in avian samples from Italy collected in 1996 [1, 2]. While initially recognized as an avian pathogen causing high mortality in blackbirds and other passerine species [2, 3], USUV has emerged as a human pathogen capable of causing severe neuroinvasive disease, particularly in immunocompromised individuals [4, 5]. The first autochthonous human infections in Europe were reported in Italy in 2009 [6, 7], and since then, cases have been documented in multiple countries across the continent [5, 8, 9]. A recent fatal infection in an immunocompromised patient in Hungary further highlights that this virus can cause severe disease [4]. USUV poses a concern for blood transfusion safety following the detection of viral RNA in asymptomatic blood donors, with positivity rates reaching up to 0.06% among Austrian blood donors in Austria during periods of high transmission [10]. Seasonal introduction of blood donor screening programs introduced in several European countries was primarily driven by concerns over West Nile virus and its transfusion transmission risk; these programs also detect USUV co-circulation [11, 12]. Multiple viral lineages currently circulate in Europe and differ in their neurovirulence [13, 14], making lineage identification important for assessing clinical and public health risks. In particular, EU2 strains exhibit high neurovirulence in animal models [14], indicating that genetic characterization of infecting strains is directly relevant for vulnerable populations such as transfusion recipients. Therefore, genomic surveillance of USUV is essential for tracking viral spread, identifying circulating lineages, and assessing public health risks. Despite its epidemiological importance, genomic surveillance of USUV in humans remains technically challenging. Blood donor samples usually contain extremely low viral loads (Ct values ~ 33–40, corresponding to ≤ 10 copies/µL), and often show partial RNA degradation due to collection and storage conditions [10, 15]. Additionally, viral RNA is greatly outnumbered by host nucleic acids in these specimens. Under such conditions, conventional metagenomic next-generation sequencing often produces fragmented viral assemblies with insufficient genome coverage for reliable phylogenetic analysis [16, 17]. Targeted amplicon sequencing protocols have revolutionized genomic surveillance for other medically important orthoflaviviruses, including Zika virus, dengue virus, and West Nile virus, by providing 10- to 100-fold increases in sensitivity through selective amplification of the viral genome [18–21]. Although targeted sequencing approaches are available for USUV, their performance declines sharply in low-titer samples and usually fails (Ct value > 32) [22–25]. As a result, genomic data from human USUV infections, especially from blood donors, remain limited, restricting insights into the diversity and spread of circulating lineages. To address this, we developed and validated a highly sensitive tiled amplicon-based sequencing protocol specifically optimized for low-titer USUV samples. This scheme covers the entire coding region of the viral genome and was designed to maximize performance on high-Ct samples. We tested the protocol using serial dilutions of reference strains and applied it to USUV-positive blood donor samples collected in Germany during the 2024 transmission season. This approach makes routine, lineage-resolved genomic surveillance of USUV in blood donor populations feasible and supports both transfusion safety monitoring and tracking of viral variants with differing pathogenicity.

## Methods

### Primer design

Altogether, 526 USUV genome sequences with > 80% completeness was downloaded from GenBank (2025-02-20) and aligned using MAFFT v7.505 [26]. Primers were designed with PrimalScheme v1.4.1 [18], targeting amplicons of ~ 250 bp. A minor modification was made to the script to increase the sequence processing limit, allowing all selected USUV sequences to be included. The final scheme consists of 65 overlapping amplicons generated by 130 primers arranged in two pools (Pool_1: 33 amplicons; Pool_2: 32 amplicons). To cover known USUV genetic diversity, 17 additional alternative primers were added, resulting in a total of 147 primers. Primers were mixed at equimolar concentrations and used at 10 µM in the multiplex PCR. The short amplicon design was chosen to improve performance on fragmented RNA. Amplicon lengths range from 241 to 262 bp, with a mean of 252.9 bp (SD ± 5.03). The scheme covers ~ 10,726 bp of the genome (positions 56 − 10,781 of reference sequence AY453412), including the complete polyprotein coding region while excluding the highly variable UTRs. Primer sequences are listed in Table S1, and the amplicon size distribution is shown in Figure S1. Primer binding was evaluated across the 526 sequences using Primer3 v3.7 [27, 28].

### In vitro isolates

USUV strains isolated from birds were propagated under Biosafety Level 3 (BSL-3) conditions at the Bernhard Nocht Institute for Tropical Medicine [29]. The viruses were grown in Vero E6 cells maintained in Dulbecco’s Modified Eagle Medium (DMEM; Lonza) at 37 °C with 5% CO₂. To establish protocol sensitivity limits, 10-fold serial dilutions were prepared from high-titer isolates representing four phylogenetically distinct lineages (strain 1477, EU3; strain 491, AFR3; strain 499, AFR2; strain 527, EU2), covering a range from approximately 5.5 × 10^6^ to 0.2 RNA copies/µL. Dilutions were prepared in DMEM and processed alongside clinical samples through the full workflow, including RNA extraction, RT-qPCR, amplicon PCR, library preparation, and sequencing. Genome coverage and sequencing depth obtained for each dilution were used to quantify the relationship between viral load and sequencing performance and to define practical thresholds for interpreting clinical samples.

### Blood donor samples

Twenty-seven USUV-positive blood donor samples were included in the study. Samples were identified during the 2024 transmission season in Germany through routine WNV screening at blood collection centers and subsequently confirmed as USUV-positive [30, 31]. Donor ages ranged from 23 to 74 years (median 59 years), including 16 males and 11 females. All donations were initially screened for WNV using a commercial nucleic acid amplification test (cobas^®^ 5800/6800/8800 systems; Roche Diagnostics). Samples that tested positive for USUV were then confirmed via USUV-specific RT-qPCR. Sample metadata, including collection date, geographic origin, and donor characteristics, are listed in Table S2.

### Nucleic acid extraction and qPCR

Nucleic acids were extracted from 140 µL of sample (cell culture supernatant or serum) using the QIAamp Viral RNA Mini Kit (Qiagen) following the manufacturer’s protocol. USUV genome copy numbers were quantified using a modified version of a previously described RT-qPCR assay [32], in which FAM fluorescence dye was replaced with Hex on the probe. Reactions were run using the OneStep RT-PCR Kit (Qiagen) with the following cycling conditions: 30 min at 50 °C, 15 min at 95 °C, followed by 45 cycles of 30 s at 95 °C, 45 s at 58 °C, and 60 s at 72 °C, with a final extension of 10 min at 72 °C. Amplification was performed on a LightCycler 480 instrument (Roche Diagnostics). Viral RNA copy numbers were calculated from Ct values using standard dilution curves.

### Usutu virus amplicon sequencing

The workflow is summarized in Figure S2. USUV-positive samples were reverse-transcribed to cDNA using SuperScript IV Reverse Transcriptase (Invitrogen). USUV-specific amplicons were generated in two parallel multiplex PCRs using Q5 High-Fidelity DNA Polymerase (New England Biolabs), with primer pools at a final concentration of 10 µM. PCR was run with an initial denaturation at 98 °C for 30 s, followed by cycles of 98 °C for 15 s and 60 °C for 1.5 min. The number of cycles was adjusted for each sample based on its Ct value (Ct rounded up to the nearest integer plus two cycles), with a maximum of 38 cycles. After amplification, products from both primer pools were combined and purified using 1.8x AMPure XP beads (Beckman Coulter, USA) with 80% ethanol washes. Amplicons were quantified, and 100 ng of DNA was used for library preparation. DNA concentrations were measured using the Qubit 1X dsDNA HS Assay Kit on a Qubit 4 fluorometer (Thermo Fisher Scientific). End repair and dA-tailing were carried out using the NEBNext Ultra II End Repair/dA-Tailing Module (New England Biolabs), followed by subsequent library preparation steps with the QIAseq FX DNA Library Kit (Qiagen, Hilden, Germany). The enzymatic fragmentation step was omitted because the amplicons were already in the appropriate size range for 300-bp read chemistry. Library fragment size was assessed using the High Sensitivity D5000 ScreenTape System, and libraries were quantified by qPCR with the KAPA Library Quantification Kit (Roche). Fragment size was also verified on a 1.5% agarose gel (SeaKem LE Agarose, Lonza) as a low-cost quality control. Normalized libraries were pooled and sequenced on an Illumina iSeq100 using 300-cycle chemistry (2 × 150 bp paired-end), aiming for 350,000-500,000 reads per sample. Negative controls were included at multiple stages of the workflow to monitor contamination, including extraction blanks, no-template controls for reverse transcription, and PCR-negative controls. The detailed protocol is available at protocols.io (https://www.protocols.io/view/usutu-virus-orthoflavivirus-usutuense-amplicon-seq-eq2lyjormlx9/v1).

### Bioinformatic analysis

Raw sequencing reads were checked for quality using FastQC v0.12.1 [33]. Paired-end reads were merged with BBMerge v38.84 [34] using default parameters (normal sensitivity) and size-selected between 230 and 270 bp to match the expected amplicon length. Primer trimming and quality filtering were performed with BBDuk v38.84 [35, 36]. For each sample, the closest available full-length USUV reference genome was identified by BLAST [37], and filtered reads were mapped to this reference using Bowtie2 v2.4.5 [38]. Consensus sequences were generated with SAMtools v1.18 [39] using a minimum coverage threshold of 20x. Read alignments and multiple sequence alignments were inspected manually in Geneious Prime v2025.0.3. Sequencing statistics for in vitro isolates and blood donor samples are provided in Tables S3 and S4. For each coverage threshold (5x, 10x and 20x), genome recovery was modeled as a function of log_10_-transform viral RNA copy number (copies/µL) using a binomial generalized linear model (GLM) with a logit link, implemented in the Python *statsmodels* package. The response variable was the fraction of the genome recovered (bounded between 0 and 1), and the predictor variable was log_10_ (copies/µL). Models were fitted using heteroscedasticity-robust (HC0) standard errors. The fitted intercept and slope were used to estimate the RNA copy number required to reach 70% genome recovery at each coverage threshold. Sequences with less than 50% genome coverage at ≥ 20x depth were excluded from downstream phylogenetic analyses.

### Metagenomic sequencing

USUV-positive blood donor samples were also analyzed by unbiased metagenomic next-generation sequencing (mNGS) to allow direct comparison with the amplicon-based approach. RNA extracted from plasma or serum was processed using an in-house mNGS workflow optimized for viral detection and sequenced on an Illumina NextSeq 2000. A detailed description of the laboratory protocol is available at protocols.io (https://www.protocols.io/view/one-health-metagenomic-next-generation-sequencing-8epv5koxnv1b/v1/metrics) [40, 41]. Raw reads were quality filtered (Phred score ≥ 20; minimum length ≥ 50 bp), and adapter sequences as well as sequence-independent single primer amplification (SISPA) primers were trimmed using CLC Genomics Workbench v22 (Qiagen, Hilden, Germany). Filtered reads were mapped to the previously identified reference genome with Bowtie2 v2.4.5, and consensus sequences were generated with SAMtools v1.18 without applying a minimum coverage threshold.

### Phylogenetic analysis

To characterize the genetic diversity and lineage assignment of USUV sequences obtained from German blood donors, we performed a comprehensive phylogenetic analysis incorporating representative sequences from all known USUV lineages. A conserved fragment of the NS5 gene, recoverable across genomes with sufficient coverage, was extracted from the assembled sequences and aligned with MAFFT v7.490 [26] together with reference sequences from lineages EU1-EU6 and AFR1-AFR4. Genomes in which this NS5 region contained extensive gaps were excluded from the analysis. Phylogenetic inference was carried out in BEAST v1.10.4 using a Bayesian Markov chain Monte Carlo framework [42]. The nucleotide substitution model was selected using IQ-TREE v2.4.0 and jModelTest v2.1.10, which identified TN93 + G + I as the best-fitting model [43–45]. Lineages were assigned based on well-supported monophyletic clades (≥ 0.7 posterior probability) following established USUV nomenclature [46]. Trees were visualized with FigTree v1.4.4 [47].

## Results

### Amplicon scheme design and coverage

The USUV amplicon sequencing scheme consists of 65 overlapping primer pairs organized in two pools (Pool 1: 33 amplicons; Pool 2: 32 amplicons) and spans 10,726 bp of the viral genome. Amplicon lengths range from 241 to 262 bp, with a mean of 252.9 bp (SD ± 5.0 bp), which allows efficient amplification from fragmented RNA typically found in clinical samples (Figure S1). Primer-template binding was evaluated in silico against a curated dataset of 526 complete USUV genomes. A primer was classified as non-binding if more than two mismatches were present or if a mismatch occurred within the first five bases at the 3′ end (Figure S3). Based on these criteria, individual primers matched 96–100% of target sites across the dataset. The mean binding rate across all primers was 99.07% (SD ± 0.96), with a median of 99.43%. Perfect primer-template matches accounted for a mean of 83.80% of binding sites (SD ± 18.66; median 92.97%). The lowest binding rate was observed for the forward primer of amplicon 18, where 3.9% of sequences were classified as non-binding. Two-mismatch binding events were rare overall (mean 0.42%, SD ± 0.72), with the highest proportion observed for the forward primer of amplicon 13 (5.32%). To improve coverage across known genetic diversity, 17 alternative primers were added to the original scheme, allowing all eight established USUV lineages to be covered. Together, these analyses show that the primer set provides broad and even coverage across lineages. As additional USUV genomes become available, the scheme can be updated by adding alternative primers at conserved binding sites within the 130 targeted primer regions.

### Evaluation of data cleaning

**Fig. 1:**
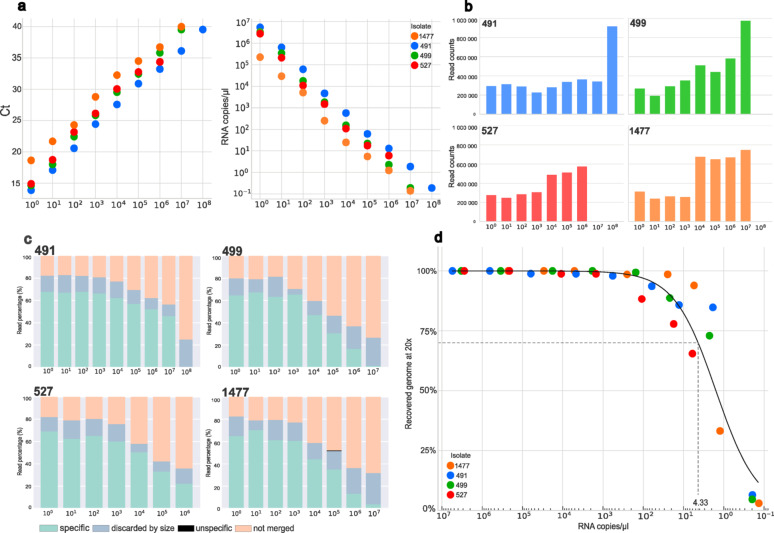
Protocol sensitivity validation across phylogenetically diverse Usutu virus lineages. (**a**) RT-qPCR performance across serial dilutions of four USUV reference strains. Scatter plots show cycle threshold (Ct) values and corresponding calculated viral copy numbers for 10-fold serial dilutions of strain 1477 (EU3), strain 491 (AFR3), strain 499 (AFR2), and strain 527 (EU2). Each dilution series spans 7–9 orders of magnitude in viral load, from high-titer stocks (> 10^6^ copies/µL; Ct ~ 14–19) to highly diluted samples approaching the stochastic detection limit (~ 0.1–0.2 copies/µL; Ct ~ 39–40). Ct values showed linear correlation with dilution across all strains; (**b**) Total sequencing read counts per dilution for strains 491, 499, 527, and 1477. Read counts ranged from ~2 × 10^5^ to 9 × 10^5^ per sample and the higher counts seen for the more dilute samples reflect the larger proportion of short primer-dimer and chimeric products in low-input libraries (which cluster efficiently and are removed during bioinformatic filtering; (**c**) Stacked bar plots showing read classification at each pipeline stage. USUV-mapped (green), size-filtered (blue), unmapped (black), and reads failing paired-end merging (orange); (**d**) Genome recovery vs. input copy number with logistic regression

**Fig. 2:**
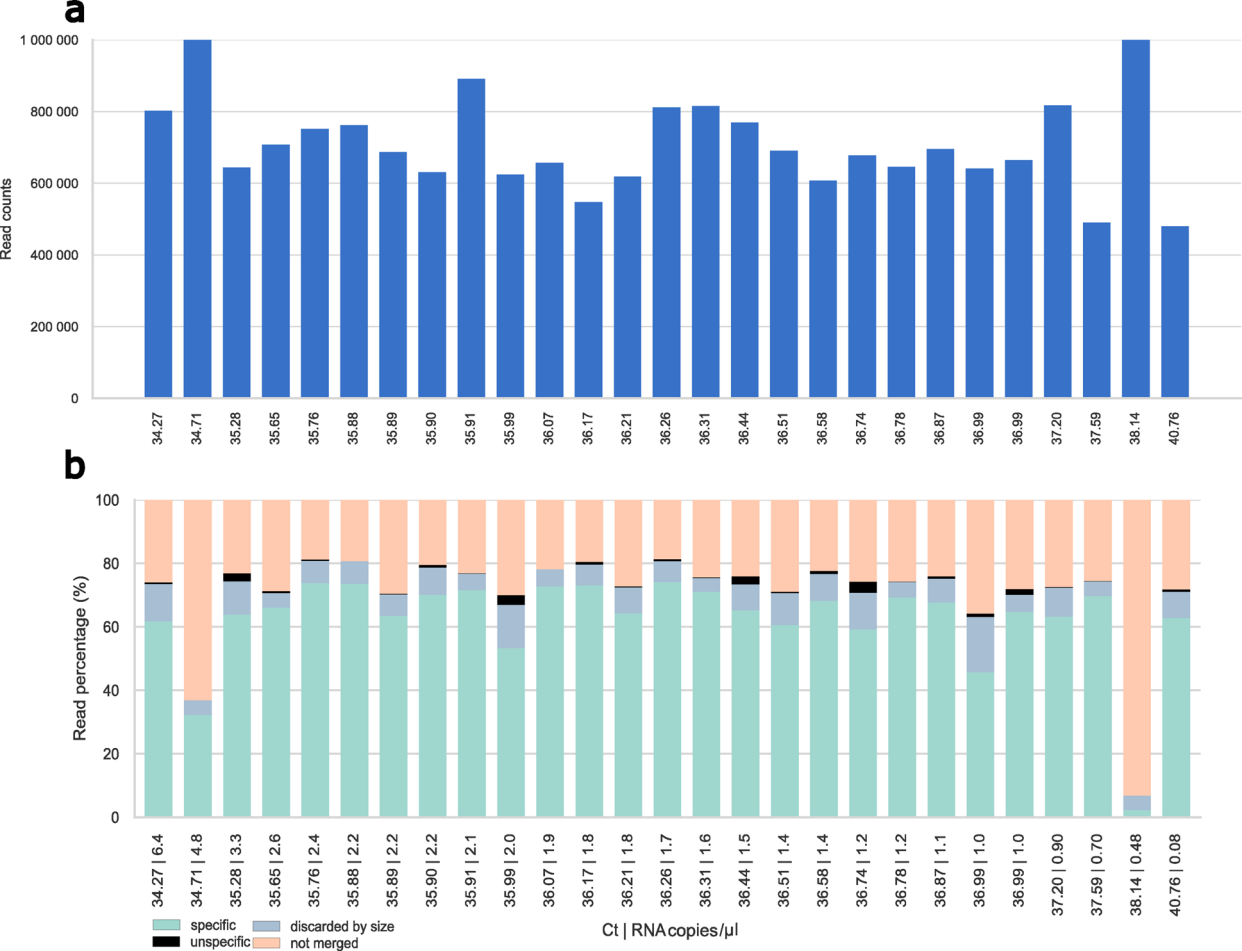
Sequencing depth and data loss in blood donor samples. (**a**) Total sequencing reads for each of the 27 blood donor samples (~ 5 × 10^5^ to ~1 × 10^6^ reads per sample); the two samples with the highest read counts, appearing at the 1,000,000-read maximum (Ct 34.71 and Ct 38.14; samples 2025_01 and 2025_03), were sequenced individually on a full flow cell and downsampled to 1,000,000 reads for downstream analyses; (**b**) Read classification through the bioinformatic pipeline. Green: USUV-mapped; blue: size-filtered; black: unmapped; orange: failed paired-end merging

Highly multiplexed short-amplicon PCR generates non-specific products (primer dimers and chimeric amplicons), which were removed bioinformatically by paired-end read merging and size-based filtering. For in vitro isolates, 17.2–75.0% of reads could not be merged (mean 36.0%; SD ± 19.3; median 14.9%), and size filtering removed a further 4.8–28.0% (mean 15.3%; SD ± 5.0). After these steps, non-specific reads (not mapping to the USUV genome) accounted for less than 0.5% in all samples (mean 0.02%; SD ± 0.09), and USUV-specific reads ranged from 0.3% to 70.3% (mean 62.3%; SD ± 15.1), increasing with viral genome copy number (Fig. 1c). Blood donor samples covered a narrower Ct range (34.27–40.76; 6.4 − 0.08 RNA copies/µL); paired-end merging removed 18.6–93.1% of reads (mean 29.0%; SD ± 15.2; median 25.7%) and size filtering a further 4.2–17.4% (mean 7.7%; SD ± 3.1; median 7.0%), non-specific reads accounted for 0.02–3.5% (mean 0.85%; SD ± 0.98; median 0.57%), and USUV-specific reads ranged from 2.35% to 74.12% (mean 62.37%; SD ± 15.10; median 65.24%; Fig. 2b). At Ct values below 30, mapped reads usually represented 50–70% of total raw reads, whereas at higher Ct values mapping rates were more variable; for example, sample 2024_30 (Ct 40.76) showed 62.8% mapped reads, whereas sample 2025_03 (Ct 38.14) showed only 2.3%. After quality filtering, mapping efficiency to the reference genome remained high (98.5–100% for serial dilutions; 94.3–99.9% for blood donor samples), and the proportion of USUV-specific reads correlated with viral copy number, with additional contributions from sample-to-sample differences in RNA quality (Figure S5).

### Sensitivity validation using serial dilutions of USUV isolates

Serial dilution experiments were conducted with cultured USUV strains 1477, 491, 499, and 527, which represent distinct phylogenetic lineages. The aim was to determine the protocol’s detection limit and to evaluate how genome recovery varies with viral load (Fig. 1; Table S3).

Sequencing depth ranged from 191,974 to 973,710 reads per sample, with a mean of 420,416 (SD ± 204,996) and a median of 323,686 reads (Fig. 1b). For strain 1477 (EU3 lineage), the undiluted sample (226,773 RNA copies/µL; Ct 18.64) achieved complete genome coverage (100% at ≥ 20x depth). High genome recovery persisted across intermediate dilutions; even dilution 1477-3 (253 copies/µL; Ct 28.79) provided coverage suitable for phylogenetic analysis (Fig. 1d; Table S3). Strain 527 (EU2 lineage) exhibited a similar pattern. The undiluted sample (2,777,686 copies/µL; Ct 14.90) also attained 100% genome coverage at ≥ 20x depth. At dilution 527-2 (10,906 copies/µL; Ct 23.17), genome coverage was 98.8% at ≥ 20x due to a single amplicon dropout, and dilution 527-4 (109 copies/µL; Ct 30.05) still achieved 88.29% coverage at the same threshold. Strain 491 (AFR3 lineage) showed comparable results; the undiluted sample (5,500,923 copies/µL; Ct 13.88) achieved complete genome recovery. Strong coverage was maintained through dilution 491-2 (61,827 copies/µL; Ct 20.58), where the first amplicon with reduced coverage (≤ 20x) emerged. Genome recovery decreased with further dilution but remained relatively high at dilution 491-7 (1.86 copies/µL; Ct 36.12), which still provided 84.7% genome coverage at ≥ 20x depth. Strain 499 (AFR2 lineage) followed the same trend, with the undiluted sample (3,306,176 copies/µL; Ct 14.64) achieving complete genome coverage, and dilution 499-4 (154 copies/µL; Ct 29.53) offering representative coverage. Across all four lineages, genome recovery stayed above 95% at ≥ 20x down to approximately 100 copies/µL (Ct ~ 30). Substantial coverage (~ 73–86% at ≥ 20x) persisted at around 10 copies/µL (Ct ~ 34–35), but recovery declined sharply below this level (Fig. 1d; Table S3). Therefore, the protocol performed well with viral loads typical of blood donor samples. For instance, dilution 491-7 (1.86 copies/µL; Ct 36.12) yielded ~ 85% genome recovery at ≥ 20x, and dilution 499-6 (2.26 copies/µL; Ct 35.83) produced 72.9% coverage at ≥ 20x, approaching the threshold for initial genomic analysis (Fig. 1; Table S3). Based on this data, the practical performance limits can be defined as consistent across all tested lineages. Samples with Ct ≤ 30 (≥ 100 copies/µL) generally produced nearly complete genomes (≥ 95% at ≥ 20x). Those with Ct 30–35 (≈ 3-100 copies/µL) yielded 65–98% coverage suitable for phylogenetic analysis, while samples with Ct 35–38 (≈ 0.2-3 copies/µL) showed variable recovery (2.7–84.7%). Samples with Ct > 38 (< 0.2 copies/µL) approached the detection limit and produced little or no usable sequence. According to GLM fits across the four strains, the viral load needed to exceed 70% genome recovery was estimated at 4.33 RNA copies/µL at ≥ 20× coverage and 2.48 RNA copies/µL at ≥ 5× coverage (Figure S4). To test whether differences in sequencing depth confounded the relationship between viral load and genome recovery, read count was additionally included as a covariate in the genome-recovery GLM, and the viral load required for 70% recovery was re-estimated with read depth held at the dataset median (Table S6). Performance was similar across all lineages, consistent with the lineage-agnostic design of the primer scheme.

### Performance in blood donor samples

Among the 27 blood donor samples, viral loads ranged from 6.43 to 0.08 RNA copies/µL (Ct 34.27–40.76), with a median of 1.70 copies/µL (Ct ~ 36.26). Two samples from the 2024 season arrived later and were sequenced individually on a full Illumina iSeq100 flow cell. Because these samples produced very high read numbers (sample 2025_01: 3,785,386 reads; sample 2025_03: 4,657,336 reads), the data were downsampled to 1,000,000 reads for downstream analyses. The target sequencing depth was 300,000-500,000 paired-end reads per sample. In total, 480,108 to 1,000,000 reads were generated per sample (mean 708,363; SD ± 123,717; Fig. 2; Table S4).

**Fig. 3:**
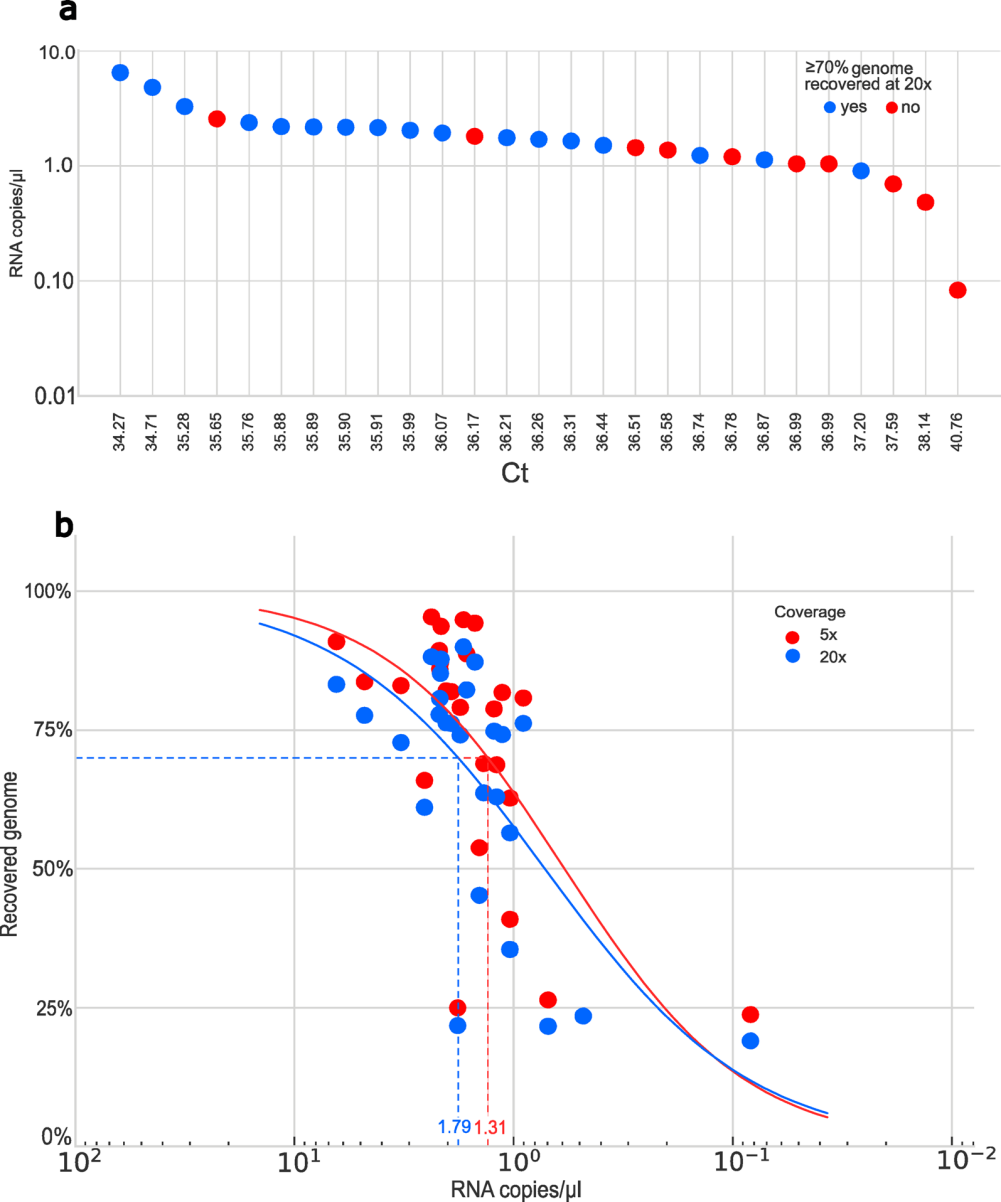
Relationship between viral load and genome recovery in blood donor samples. (**a**) Samples ranked by viral load (RNA copies/µL) showing whether ≥ 70% of the genome was recovered at ≥ 20x coverage. (**b**) Scatter plot depicting the association between viral load (RNA copies/µL; log scale) and the percentage of genome recovered for all 27 blood donor samples at 5x (red) and 20x (blue) coverage. Solid curves represent fitted logistic regression models for each coverage depth. Horizontal dashed line indicates the ≥ 70% genome recovery threshold, and vertical dashed lines denote the estimated viral load cutoffs corresponding to this threshold at 5x and 20x coverage, respectively

Genome recovery at ≥ 20x coverage ranged from 18.9% to 89.9% and was slightly higher at ≥ 10x depth (21.3–92.5%). Previous orthoflavivirus genomic epidemiology studies indicate that recovery of at least 70% of the genome provides sufficient phylogenetic signal for reliable subclade assignment and phylogeographic reconstruction [48, 49]. Using this cutoff, 17 of the 27 blood donor samples (63%) achieved ≥ 70% genome coverage at ≥ 20x depth and were therefore suitable for lineage assignment across the coding region (Fig. 3).

Even the lowest-titer sample (2024_30; 0.08 RNA copies/µL; Ct 40.76) yielded 18.9% genome coverage at ≥ 20x depth. Among samples with viral loads above 1.5 RNA copies/µL (*n* = 16), 14 reached ≥ 70% genome recovery at ≥ 20x. Two samples (2024_89 and 2024_60) showed lower than expected coverage despite viral loads of 2.55 and 1.80 copies/µL, yielding 61.0% and 21.7% genome recovery, respectively (Table S4). Samples with viral loads below 1.5 copies/µL were more variable but still produced substantial coverage (18.9–76.1%; Fig. 3). No systematic amplicon dropouts were detected in low-titer samples. Instead, coverage gaps were scattered across the genome, consistent with stochastic sampling rather than primer failure or contamination. The ≥ 70% genome recovery threshold closely matched the values predicted from the serial dilution experiments. Based on modeling of blood donor data, the viral load required to exceed 70% genome recovery was estimated to be 1.79 RNA copies/µL at ≥ 20x coverage and 1.31 RNA copies/µL at ≥ 5x coverage (Fig. 3; Table S4).

### The relation between the viral load and genome recovery

Analysis of all 59 samples (32 serial dilutions from four reference strains and 27 blood donor samples) revealed a strong dependence of genome recovery on viral RNA concentration. This relationship was observed for all USUV lineages included in the study, showing that the performance of the amplicon scheme was not lineage-dependent. In blood donor samples, coverage gaps did not recur at the same genomic locations but were scattered across the coding region (Figure S4-6), consistent with stochastic loss rather than primer failure. Multiple amplicons were recovered from every USUV-positive sample, while all negative controls yielded no amplicon reads, fulfilling standard criteria for detection specificity and excluding cross-contamination.

**Fig. 4:**
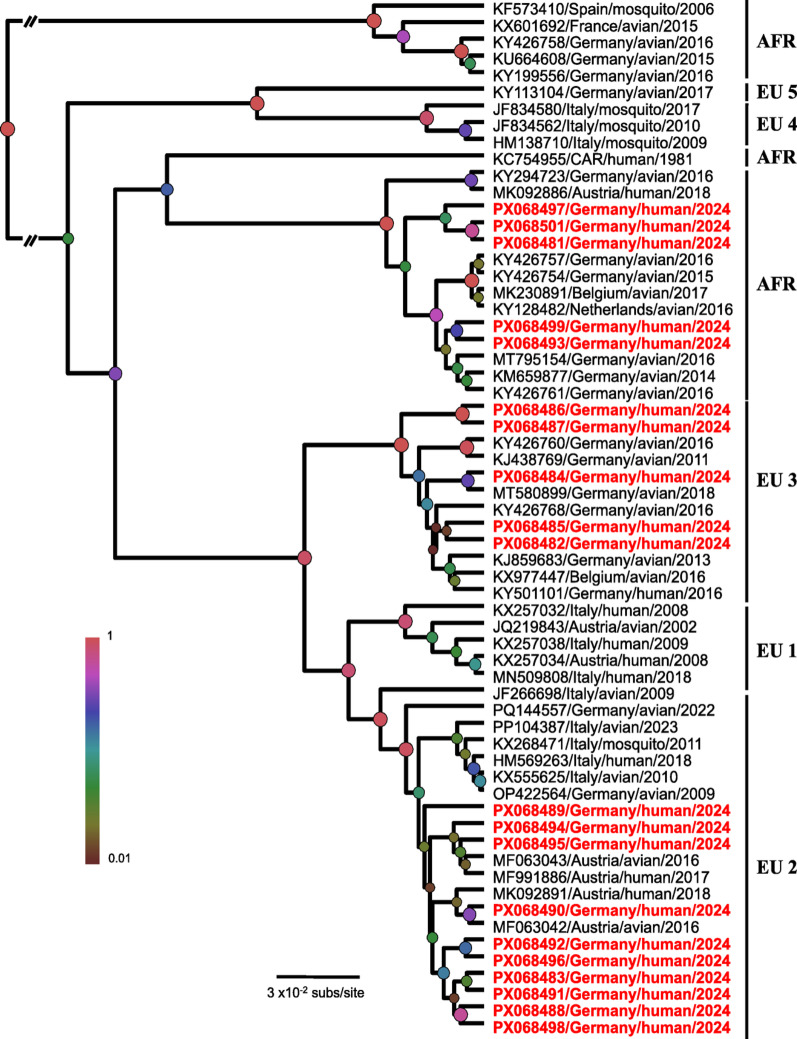
Bayesian maximum clade credibility (MCC) phylogenetic tree of USUV. The tree shows the placement of human Usutu virus cases detected in this study (red) in the context of representative USUV strains. The tree was reconstructed using NS5 gene sequences and inferred with a Bayesian Markov chain Monte Carlo (MCMC) approach implemented in BEAST v1.10.0. Node support values correspond to Bayesian posterior probabilities (clade credibility) and are indicated by colored circles at the respective nodes. GenBank accession numbers, country of origin, host species, and year of detection for each sequence are displayed at the branch tips. The scale bar represents the mean number of nucleotide substitutions per site

### Comparison of targeted and metagenomic approaches

In parallel, blood donor samples were also analyzed by unbiased viral metagenomic next-generation sequencing (mNGS). Sequencing depth ranged from 3,674,118 to 12,535,258 single reads per sample (SD ± 2,963,220; Figure S7). Five of the 27 samples were negative for USUV by metagenomic analysis. Across all samples, the number of USUV reads ranged from 0 to 416, corresponding to 0-43.8 reads per million. Because USUV reads were sparse, ≥ 20x genome coverage was reached in only three samples. Genome recovery ranged from 0 to 29.7% at a ≥5x coverage threshold and increased to a maximum of 54.0% when a ≥1x threshold was used (Figure S7; Table S5). The amplicon-based approach yielded higher genome recovery than metagenomic sequencing for all samples except 2024_30, for which metagenomics produced slightly more coverage (27.2%) than the targeted approach (24.3%). None of the samples reached 70% genome recovery by metagenomic sequencing, even at a ≥1x coverage threshold. In addition, the amplicon approach required much lower sequencing depth. Targeted sequencing generated a mean of 708,364 single reads per sample (median 686,786), compared with 9,305,702 reads (median 10,233,514) for metagenomic sequencing. Overall, the amplicon-based method is more suitable for genome recovery from low-titer blood donor samples, while metagenomic sequencing remains valuable for diagnostic detection, even at very low viral loads (< 10 RNA copies/µL), but is insufficient for high-resolution genomic characterization in this setting.

### Phylogenetic characterization of blood donor infections

To assess whether the amplicon-based data were suitable for phylogenetic analysis and lineage assignment, we analyzed 20 blood donor-derived USUV genomes with ≥ 50% genome recovery together with reference sequences representing all established USUV lineages. The resulting phylogeny showed that the sequences had sufficient resolution for reliable lineage assignment and diversity analysis (Fig. 4). Several USUV lineages were detected among German blood donors during the 2024 season, including EU2, EU3, and AFR3. All blood donor sequences were grouped within well-supported monophyletic clades (Bayesian posterior probability > 0.95), confirming both sequence quality and phylogenetic informativeness. Within each lineage, the German blood donor sequences showed little genetic variation (mean pairwise nucleotide distance < 0.1%), consistent with recent circulation from closely related sources during the 2024 season. Despite the limited number of samples, differences in lineage distribution were apparent. In eastern Germany (Brandenburg and Berlin), only EU2 was detected. In northern Germany (Schleswig-Holstein), both EU2 and AFR3 were present, whereas samples from southwestern Germany (Rhineland-Palatinate, Baden-Württemberg, and Hesse) showed co-circulation of AFR3 and EU3. A single sequenced sample from North Rhine-Westphalia belonged to EU3 (Figure S8). While these patterns point to regional variation in circulating lineages, additional genomic data will be needed to resolve spatial and temporal transmission dynamics with confidence.

## Discussion

Over the past three decades, USUV has spread widely across Europe, with sustained transmission in multiple countries and infection of a broad range of hosts. These patterns indicate that USUV represents an emerging transcontinental public health risk with relevance for both Europe and Africa [50–52]. Understanding how USUV spreads and evolves requires a One Health perspective supported by reliable genome sequence data [53]. For West Nile virus, major gaps in African genomic surveillance are well known, and similar deficiencies exist for USUV [54]. Generating more sequence data from both continents is therefore essential for tracking viral diversity and transmission. Amplicon-based targeted sequencing approaches are valuable tools to generate specific sequence data. This approach has already proven useful during outbreaks of several viral pathogens [20, 48, 55] and offers faster turnaround times and lower costs than capture-based (bait hybridization) enrichment methods [56–58]. Although capture approaches are theoretically more flexible in target selection, tiled amplicon sequencing can be rapidly adapted to newly emerging or neglected viruses through updated primer design and bioinformatic workflows. The use of random hexamer priming during reverse transcription helps accommodate sequence variation across divergent lineages, which is particularly important for surveillance settings where unexpected variants may be encountered. The Q5 high-fidelity polymerase used here has a low intrinsic error rate, and high sequencing depth allows errors to be corrected during consensus generation. Low-frequency variants in low-titer samples should nevertheless be interpreted cautiously and, where relevant, confirmed by independent amplification. The combination of short amplicons and high-cycle PCR (up to 38 cycles) allows sufficient sensitivity to detect samples containing roughly 10–100 RNA copies/µL, even though reverse transcription specificity is reduced at such low input levels. This approach builds on tiled-amplicon strategies developed for other medically important flaviviruses, including Zika virus [18], West Nile virus [59], and dengue virus [20], which have shown much higher sensitivity than metagenomic sequencing for low-titer clinical samples. Although several USUV-specific amplicon protocols have been described, their sensitivity often falls short of enabling full genome recovery at low viral loads (~ 10–100 RNA copies/µL), and many remain either insufficiently validated or not broadly accessible [22–25]. Here, we present a validated amplicon-based sequencing protocol designed for high-sensitivity genomic surveillance of USUV, including blood donor samples. Genome recovery of at least 70% was used as the criterion for successful sequencing. Although lineage assignment itself can be achieved from a conserved NS5 fragment, near-complete genome recovery is required for the broader applications which support genomic surveillance, genomic epidemiology and phylogeography, molecular-clock dating, and detection of variants, recombination and virulence determinants distributed across the genome, none of which can be resolved from a single gene [20, 48, 60]. Serial dilution experiments defined practical performance thresholds: samples with Ct ≤ 30 (≥ 100 copies/µL) yielded near-complete genomes (≥ 95% at 20x depth); Ct 30–35 (3-100 copies/µL) achieved 65–94% coverage suitable for phylogenetic analysis; and Ct 35–40 (0.1-3 copies/µL) produced variable results (2–85%), becoming increasingly unpredictable above Ct 37. When applied to 27 USUV-positive blood donor samples from Germany, the protocol enabled reliable lineage assignment and high-resolution phylogenetic analysis for most specimens. Robust genome recovery (≥ 70% at 20x) was achieved for 63% of samples at viral loads as low as 0.08–6.43 RNA copies/µL (median 1.69 copies/µL), a range that is typically not accessible to metagenomic sequencing. Performance was consistent across four phylogenetically distinct lineages (EU2-EU3 and AFR2-AFR3), supporting the broad applicability of the approach. Several design features contributed to this sensitivity. The tiled scheme uses 65 short amplicons (241–262 bp; mean 252.9 bp), which improves performance on fragmented RNA from stored clinical samples. Illumina sequencing was chosen over nanopore because of its higher raw read accuracy, which supports reliable consensus calling and detection of intrahost variation in low-titer samples [61]. The high degree of multiplexing leads to the formation of primer dimers and chimeric reads (mean 36.7%), but these were effectively removed by paired-end read merging and size filtering. Because chimeric products longer than 300 bp cannot be merged by the 2 × 150-bp chemistry, and because non-specific products increase at low template input when only a few viral RNA molecules enter reverse transcription and the first PCR cycles, Poisson sampling, early priming events and differences in RNA integrity determine which templates are amplified mapping rates were more variable at high Ct values, although on-target read fractions remained generally sufficient across samples spanning four orders of magnitude in viral load. This short-amplicon scheme therefore requires more reads than tiled-amplicon protocols with longer amplicons (~ 400), such as the ARTIC SARS-CoV-2 scheme, particularly for low-titre samples. Based on the validation experiments, about 300,000 paired-end reads were sufficient for complete genome recovery from samples with Ct < 30, whereas samples with Ct ≥ 30 generally required around 500,000 reads. These capabilities address a major gap in USUV surveillance. Detection of USUV RNA in asymptomatic blood donors has raised concerns for transfusion safety, with positivity rates reaching 0.06% in Austria during peak transmission periods and seasonal screening [10]. In Germany, a low but increasing frequency of USUV-positive blood donations was observed between 2022 and 2024 [30]. However, the very low viral loads in these samples (Ct 33–40) have so far limited insight into circulating lineage diversity and geographic transmission dynamics. Using the approach described here, all known USUV lineages associated with human infection in Germany (EU2-EU3 and AFR3) could be reliably characterized from blood donor material [62]. Like West Nile virus, USUV infections in immunocompetent individuals are typically associated with short-lived, low-level viremia. The protocol can therefore also be applied to clinical cases of USUV disease, such as meningitis or encephalitis, although further validation with additional sample types (e.g., whole blood, urine, or tissue) will be required. Given experimental evidence for lineage-dependent differences in neurovirulence [14, 63], broader genomic surveillance of both symptomatic and asymptomatic infections will be important to better understand disease severity and pathogenesis. Recent fatal USUV infections in immunocompromised patients further emphasize the clinical relevance of transfusion-transmitted infections [4, 64, 65]. The Ct-based performance thresholds established here allow practical sample triaging in surveillance programs. Samples with Ct ≤ 35, which are expected to yield at least 65% genome coverage, can be prioritized for sequencing, while samples with Ct 35–38 may be sequenced selectively when specific epidemiological questions arise. Routine sequencing of samples with Ct > 38 is unlikely to be informative because of strong stochastic effects. With a turnaround time of approximately 1.5 days from sample receipt to consensus sequence, the protocol supports near-real-time genomic surveillance. At current sequencing costs and success rates, this approach is also economically sustainable, in contrast to metagenomic sequencing, which requires much higher read numbers and yields lower success for high-Ct samples. Several limitations should be noted. Below about 1 RNA copy/µL (Ct ~ 37), stochastic sampling effects impose a fundamental limit on genome recovery. Sensitivity could potentially be improved by increasing extraction volumes or concentrating on RNA, but this would add complexity. The primer design targets currently known European and African lineages, and substantial divergence at primer binding sites in future variants could reduce amplification efficiency. Regular primer updates incorporating newly available sequences will maintain broad reactivity. High numbers of PCR cycles may also introduce replication errors, particularly in very low-titer samples. Overall, this study shows that systematic genomic surveillance of USUV in blood donor populations is now technically and economically feasible using targeted amplicon sequencing. The protocol supports lineage-specific risk assessment, monitoring of viral spread, and detection of new variants, which are all important for blood safety and public-health preparedness. With demonstrated success on blood donor samples, the approach transforms previously intractable clinical specimens into sources of actionable genomic intelligence. As USUV continues to expand in Europe and human exposure increases, the genomic surveillance capacity established here provides an important tool for managing this emerging neurotropic virus [66].

## Supplementary Information

Supplementary Material 1.

Supplementary Material 2.

## Data Availability

Raw sequencing data have been deposited in the NCBI Sequence Read Archive under BioProject accession PRJNA1289158. Consensus USUV genome sequences with ≥50% genome recovery have been deposited in NCBI GenBank. Individual sample accession numbers are provided in Tables S3 and S4.
