# Supplementary material for: A highly sensitive amplicon sequencing workflow for genomic surveillance of Usutu virus
Source: Virol J. 2026 Jul 10;23:174. doi: 10.1186/s12985-026-03251-w (PMC13352883; doi:10.1186/s12985-026-03251-w)

**Supplementary Figures**

**Supplementary Figure S1.** Usutu virus amplicon scheme design and characteristics. **(a)** Overview of the developed USUV amplicon sequencing scheme consisting of 65 overlapping amplicons distributed across two primer pools. Bars represent individual amplicons and are colored by primer pool (pool 1, blue; n=33 amplicons; pool 2, orange; n=32 amplicons). Amplicon lengths ranged from 241 to 262 bp (mean 252.9 bp ± 5.0 SD) and spanned 10,726 bp of the viral genome, covering the complete coding region. **(b)** Amplicon length distribution across the full scheme. The histogram displays the size distribution of all 65 amplicons, showing the amplicon grouping close to the target length of ~250 bp.

**Supplementary Figure S2.** Overview of the Usutu virus amplicon-based sequencing and bioinformatic analysis workflow. The schematic illustrates the complete laboratory and computational pipeline from sample processing to phylogenetic analysis. Created with Biorender.com.


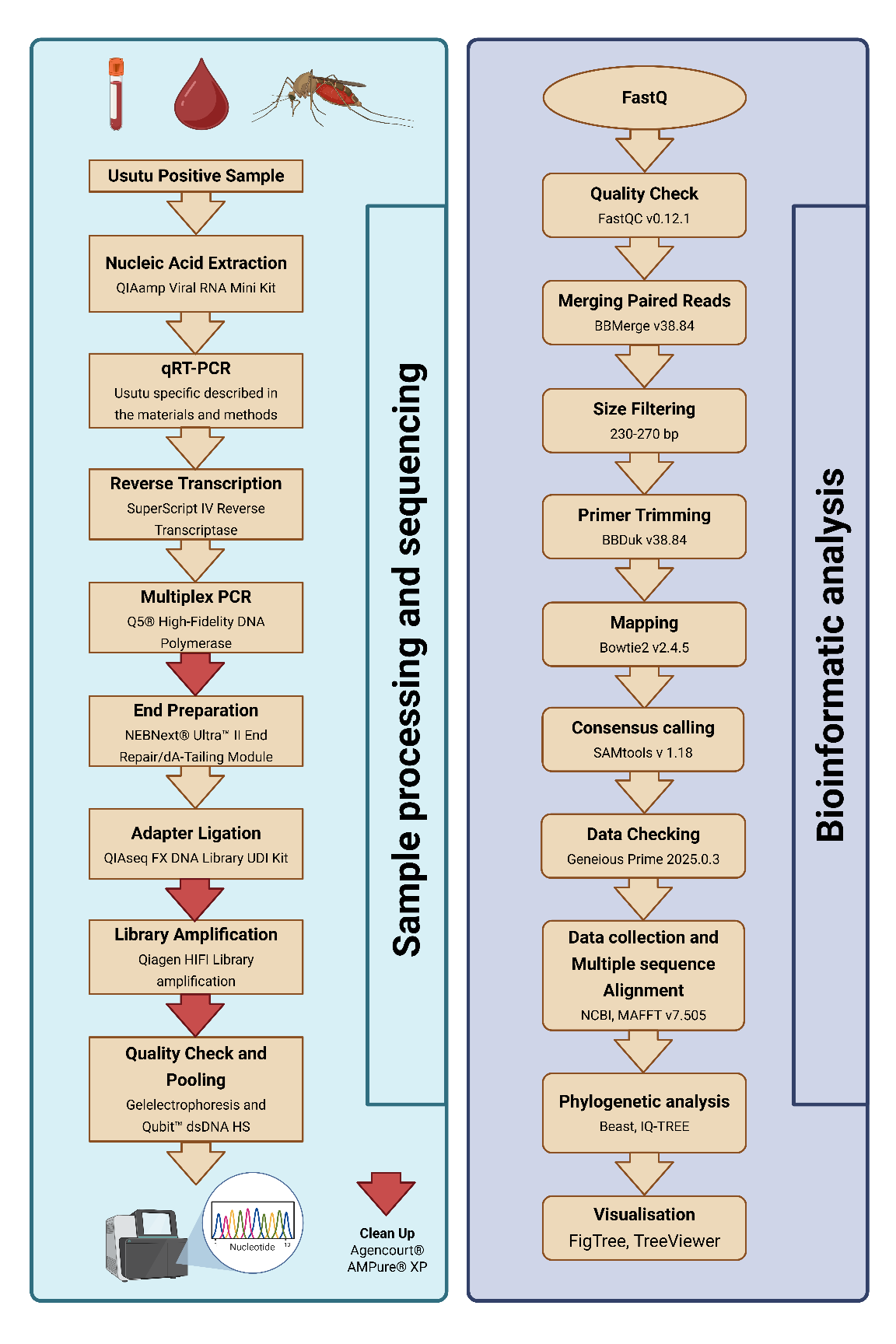


**Supplementary Figure S3.** In silico primer binding across the USUV amplicon sequencing scheme. Horizontal stacked bar plots show the proportion of reference USUV genomes with perfect primer binding (green), one mismatch (light blue), two mismatches (black), or no predicted binding (orange) for each forward (F) and reverse (R) primer included in the scheme. Primer identifiers are shown on the y-axis, and the x-axis indicates the percentage of binding sites across all analyzed USUV reference sequences.


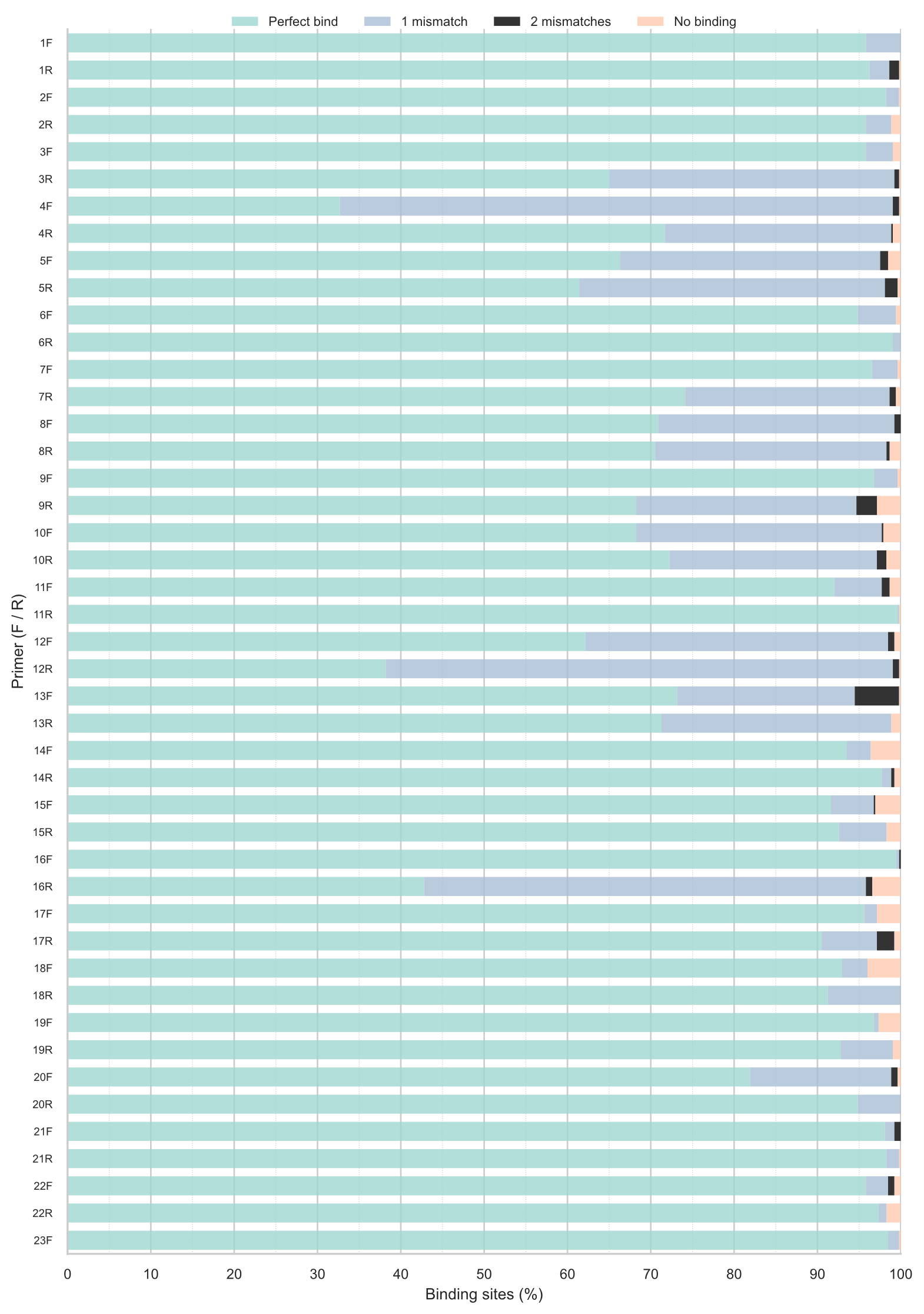


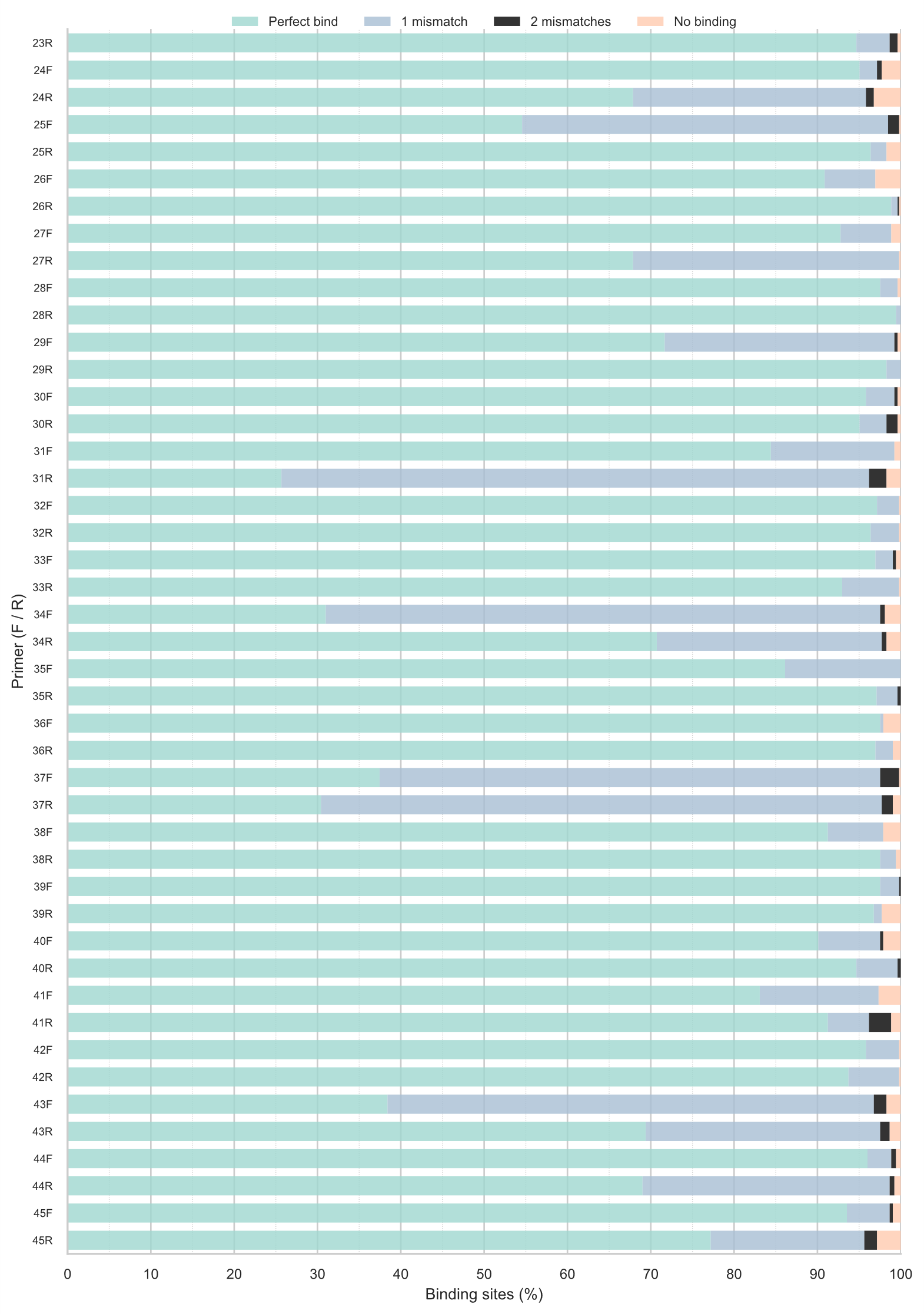


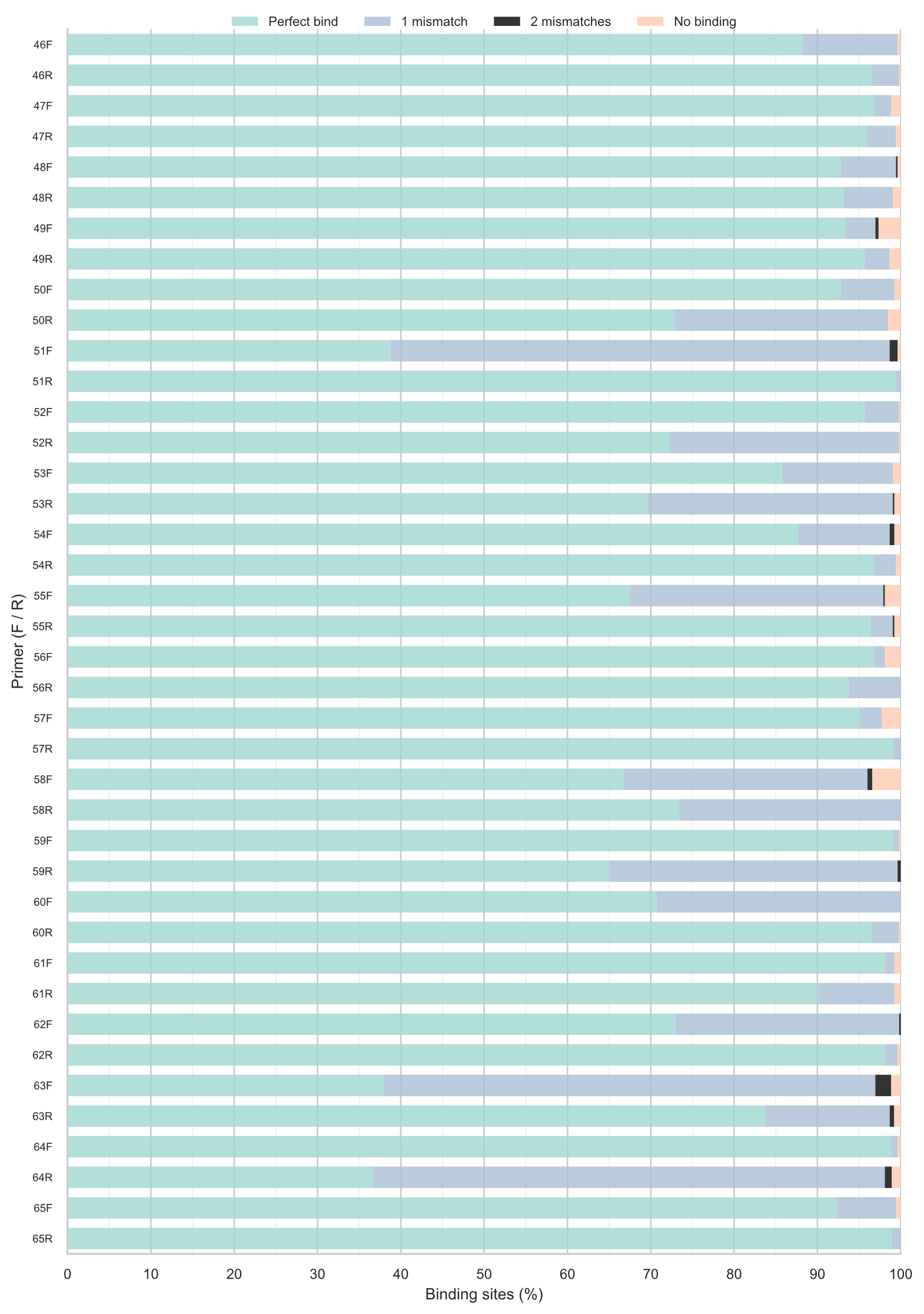


**Supplementary Figure S4.** Viral load and genome recovery across USUV reference strains. Scatter plots show the proportion of the genome recovered (y-axis) as a function of viral RNA concentration (copies/µL; log_10_ scale on the x-axis) at ≥5x (red) and ≥20x (blue) sequencing coverage. Solid lines indicate fitted logistic regression models for each coverage threshold. Horizontal dashed lines mark the 70% genome recovery threshold, and vertical dashed lines indicate the corresponding viral RNA concentrations (4.33 RNA copies/µL at ≥20x and 2.48 RNA copies/µL at ≥5x coverage). Points represent serial dilutions of four phylogenetically distinct USUV lineages (EU2, EU3, AFR2, and AFR3)

**Supplementary Figure S5.** RT-qPCR Ct values and the proportion of USUV-specific sequencing reads. (a) Serial dilution experiments using USUV reference strains. Scatter plot showing the percentage of reads mapping to the USUV genome (y-axis) as a function of Ct value (x-axis) for strains 491 (AFR3), 499 (AFR2), 527 (EU2), and 1477 (EU3). Each point represents a single dilution. The grey line shows a fitted regression across all isolates. (b) Blood donor samples Scatter plot shows the percentage of reads mapping to the USUV genome (y-axis) plotted against Ct value (x-axis) for all USUV-positive blood donor samples. Each point represents one sample, and the line indicates a fitted regression.

**a**

**b**

**Supplementary Figure S6.** Amplicon-based genome coverage in USUV-positive blood donor samples. Genome coverage depth across the viral genome is shown for selected blood donor samples sequenced with the amplicon-based approach. Each horizontal panel corresponds to a single sample (sample IDs shown on the left). Genome position (bp) is plotted on the x-axis and read depth on the y-axis (log_10_ scale). Bars indicate coverage for individual amplicons.

**Supplementary Figure S7.** Unbiased metagenomic sequencing of USUV-positive blood donor samples. (a) Total raw sequencing read counts generated per sample by unbiased viral metagenomic next-generation sequencing (mNGS). Read counts ranged from approximately 3.7 to 12.5 million reads per sample. (b) Percentage of the USUV genome recovered (y-axis) plotted against viral RNA concentration (copies/µL; log_10_ scale on the x-axis) using ≥1x (red) and ≥5x (blue) coverage thresholds. Each point represents an individual blood donor sample. The dashed horizontal line marks the 70% genome recovery threshold.

**a**

**b**

**Supplementary Figure S8.** Geographic distribution of USUV-positive blood donor samples in Germany. Sampling locations are shown as colored points on a map of Germany. Colors indicate the inferred viral lineage based on phylogenetic analysis (AFR3, green; EU2, orange; EU3, red). Major cities are shown for geographic reference.


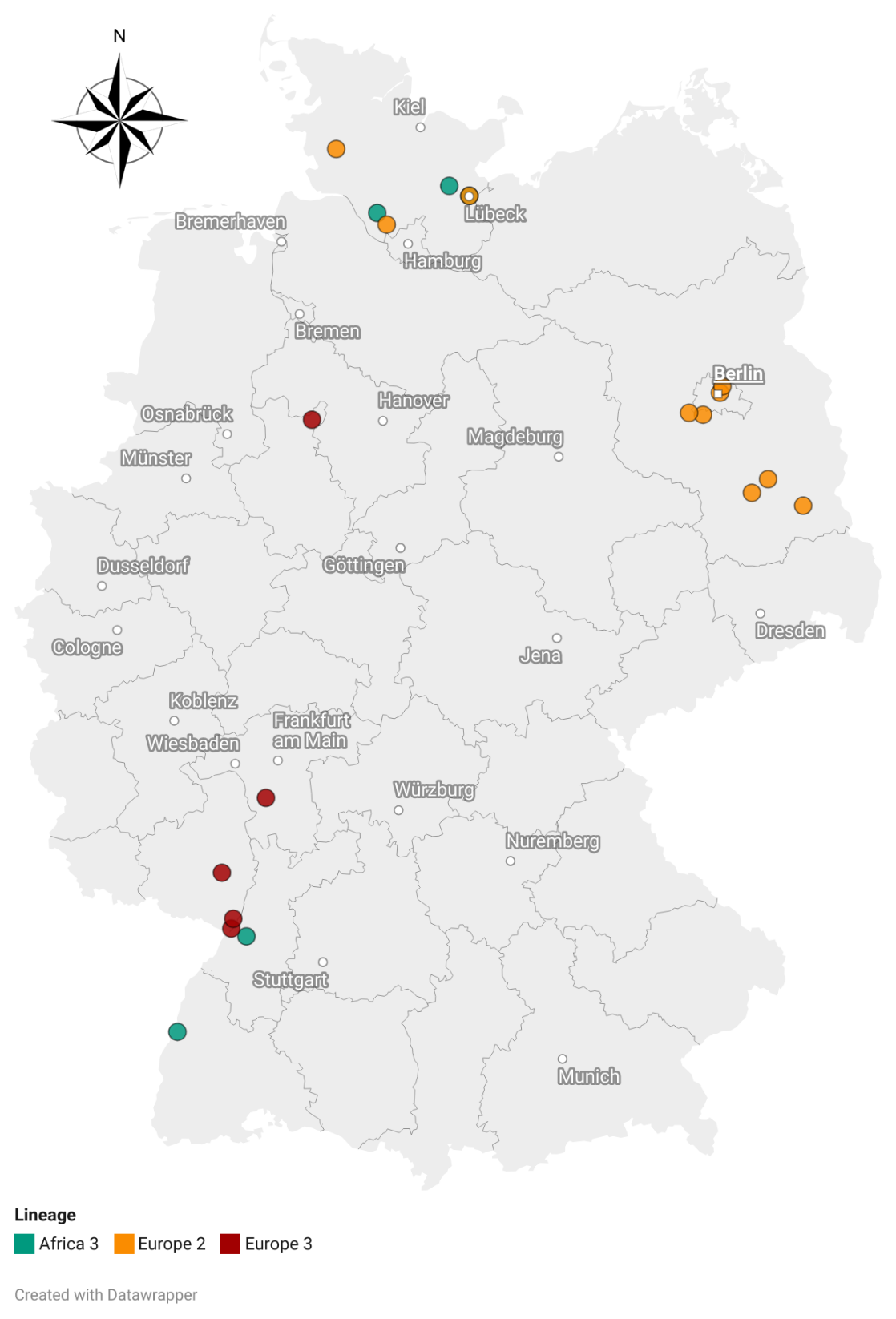

Supplement: Supplementary file 2 — Supplementary Material 2. [file 12985_2026_3251_MOESM2_ESM.docx]
